# A 3D-Printed Bent–Twisted Waveguide Filter Using Mixed TE_101_ and TE_102_ Mode Resonators

**DOI:** 10.3390/mi16030247

**Published:** 2025-02-21

**Authors:** Lei Wang, Mengke Bai, Jun Xu

**Affiliations:** 1 The 54th Research Institute of CETC, Shijiazhuang 050081, China; wl198211042025@163.com; 2 School of Physics, University of Electronic Science and Technology of China, Chengdu 611731, China; xujun@uestc.edu.cn

**Keywords:** 3D printing, waveguide bandpass filter, bent–twisted waveguide filter

## Abstract

This paper presents a waveguide bandpass filter that integrates bending and twisting functions, utilizing mixed TE_101_ and TE_102_ mode resonators. Benefiting from the mixed utilization of TE_101_ and TE_102_ resonators, the desired stopband suppression and low insertion loss are achieved. To verify the feasibility of this approach, a sixth-order Chebyshev bandpass filter centered at 20.4 GHz is designed, with a bandwidth of 1.6 GHz (FBW = 7.84%) and a return loss of more than 20 dB. The filter also achieves 120° bending in the propagation direction and 90° rotation in polarization. This prototype is fabricated following the stereolithography (SLA) process using photosensitive resin, followed by metallization through electroplating to achieve a lightweight design. The measurements exhibit great consistency with the simulations.

## 1. Introduction

Waveguide bandpass filters (BPFs) are essential components in communication systems, offering low insertion loss, high power handling capacity, and great frequency selectivity. However, with the rapid advancement of wireless communication technology, traditional waveguide filters face significant challenges, particularly in terms of size and weight. To address these challenges, key trends in filter design have shifted towards miniaturization, integration, lightweight structures, and multifunctionality [1,2,3,4,5]. In response to these evolving demands, researchers have proposed more complex filter structures, such as irregular resonators, novel coupling schemes, and flexible waveguide designs. Despite their potential, these designs often present fabrication challenges, especially when using traditional Computer Numerical Control (CNC) techniques. In this context, 3D printing has emerged as a promising fabrication solution. By enabling the creation of monolithic structures, 3D printing eliminates the need for complex cutting and milling processes, offering a more streamlined approach to manufacturing. A variety of 3D-printed waveguide BPFs have been proposed in recent years, and filters based on spherical resonators are one of the most widely researched fields [6,7,8,9,10,11]. Spherical resonators have higher unloaded quality factors than rectangular cavities, contributing to a lower insertion loss. However, the high symmetry of the spherical structure leads to resonant frequencies of higher-order modes becoming more closely spaced, thereby reducing stopband rejection. On some occasions, short slots are added to the cavity walls to suppress the harmonic response of undesired modes [12]. Further, hemisphere and elliptical resonators have also been proposed [12,13,14].

Waveguide BPFs that integrate bending and twisting functions have also been extensively researched and tested by 3D printing. They have compact configurations and high flexibility in communication systems. Traditionally, the design methods aim to cascade waveguide bends or twists with BPFs, resulting in a large volume and an inevitable deterioration in insertion loss. An advanced solution provides a higher integration and better RF performance, which directly applies rotation to the structures of BPFs, to achieve filtering and bending or twisting simultaneously. Notable studies include a lowpass filter with a 90° bend and a 90° twist [15], two 90°-twisted waveguide BPFs utilizing step-twisted rectangular waveguide resonators [16] and gradually twisted ones [17], and a 90° bent–twisted BPF using gradually distorted rectangular waveguide resonators [18]. And, in [14], BPFs based on elliptical or ellipsoidal resonators are proposed to achieve more flexible waveguide routines.

Moreover, gap waveguide is an advanced technology to realize integration and miniaturization as well, and 3D-printed gap waveguide BPFs are proposed in [19,20]. They have a quasi-planner structure and great performance comparable to those of traditional waveguides.

On the other hand, mixed mode technology is also widely used in filter designs, which mainly improves the suppression of harmonic response and introduces transmission zeroes. In [21], BPFs with transmission zeroes based on mixed TM_110_ and TE_10½_ modes is proposed. In [22], a BPF with a wide stopband is proposed by using mixed substrate-integrated cavities. In [23], a dual band BPF using mixed TE_301_ and TE_102_ modes was proposed, with a high quality factor of the cavities and insensitivity in fabrication. And, in [24], an inverted microstrip gap waveguide BPF with high selectivity was proposed, which perturbs fields of TE_102_ and TE_103_ modes to obtain a hybrid cavity and utilizes another microstrip stub-loaded resonator mode in order to obtain a total of four transmission zeroes. Moreover, mixed mode applications including filtering antennas [25], diplexers [23], and crossovers [26] are also researched.

**Figure 1:**
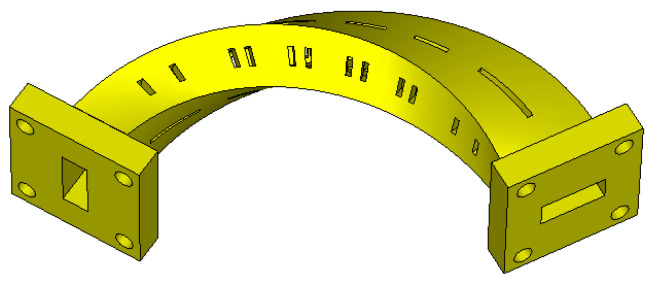
Overall configuration of the proposed BPF.

This paper presents a waveguide bandpass filter (BPF) that includes both TE_101_ and TE_102_ resonators, integrated with a 90° polarization twist and a 120° bend in the propagation direction. A photograph of the overall configuration is shown in Figure 1. The filter exhibits lower insertion loss due to the larger volume of the TE_102_ cavities, which offer a higher unloaded quality factor (*Q_u_*) than the TE_101_ cavities. Additionally, compared to BPFs using only TE_102_ cavities, it demonstrates better stopband performance. To validate this concept, a filter prototype is designed and then fabricated by the SLA process, one of the most mature 3D printing technologies that uses photosensitive resin as its material. The measured insertion loss is about 0.46 dB on average and lower than 0.94 dB, while the return loss is better than 10.37 dB. The little shift in the center frequency of about 100 MHZ (0.5%), toward higher frequencies, shows great consistence with the simulations. Finally, the proposed BPF is compared with the other three bent–twisted BPFs [16,17,18] proposed previously to illustrate its novelty and advantages, including the mixed use of the TE_101_ and TE_102_ modes, a lower insertion loss, a novel 120° bend in the waveguide routine, and better selectivity.

## 2. Analysis of the Resonator

**Figure 2:**
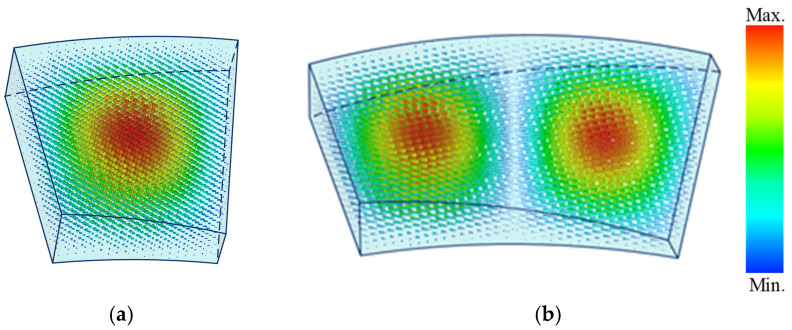
Electric fields of distorted waveguide cavities: (**a**) TE_101_ mode cavity; (**b**) TE_102_ mode cavity.

**Figure 3:**
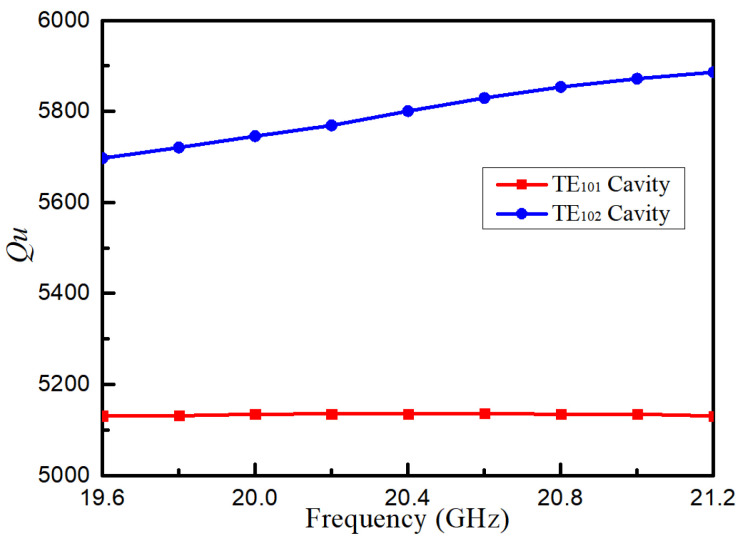
Unloaded quality factors *Qu* of TE_101_ mode and TE_102_ mode cavities.

The two types of cavities used in the filter are shown in Figure 2, along with the simulated electric fields from EM simulation software. Distorted rectangular waveguide resonators are utilized to complete the gradual twisting and bending while maintaining similar electromagnetic fields to those of standard rectangular cavities. The resonant frequency of the TE_101_ mode and TE_102_ mode in a distorted rectangular waveguide resonator can be approximately calculated using the following equations:

(1) $${f_{\mathrm{TE}}}_{101}\sim \frac{c}{2}\sqrt{{\left(\frac{1}{a}\right)}^{2}+{\left(\frac{1}{\theta r}\right)}^{2}}$$

(2) $$f_{{\mathrm{TE}}_{102}}\sim \frac{c}{2}\sqrt{{\left(\frac{1}{a}\right)}^{2}+{\left(\frac{2}{\theta r}\right)}^{2}}$$

where *c* = 3 × 10^8^ m/s is the speed of light in a vacuum, *a* is the width of the waveguide, *θ* is the bending angle, and *r* is the bending radius. The unloaded quality factors *Q_u_* of the two types of cavities are pictured in Figure 3. The cavity walls are assumed to be made of copper, with electrical conductivity at 5.96 × 10^7^ S/m. A higher *Q_u_* of the TE_102_ cavity can be observed, which resulted from a larger volume, and consequently contributes to a lower insertion loss.

**Figure 4:**
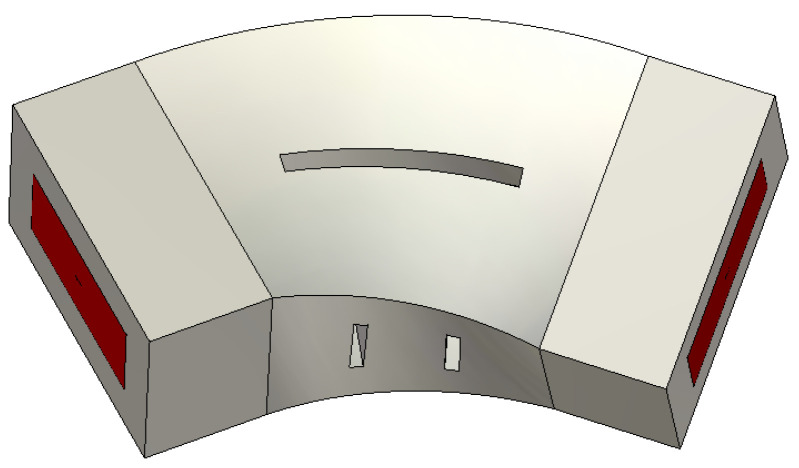
The doubly loaded TE_102_ resonator.

In the actual resonator design, rectangular slots are introduced into the cavity walls to facilitate the subsequent electroplating process and ensure smoother flow of the plating solution into the waveguides, as shown in Figure 1. The orientations of the slots are along the surface currents to miniaturize the radiation and the disturbance to electromagnetic fields. To demonstrate that the additional radiation loss is negligible, an EM simulation of the resonator is carried out and quality factors are analyzed by the equations

(3) $$Q_{L}^{-1}=Q_{e}^{-1}+Q_{c}^{-1}+Q_{r}^{-1}$$

(4) $$Q_{u}^{-1}=Q_{c}^{-1}+Q_{r}^{-1}$$

where *Q_L_*, *Q_e_*, *Q_c_*, and *Q_r_* represent the loaded, external, conductor, and radiation quality factors, respectively. *Q_L_* can be calculated using the following equation:

(5) $$Q_{L}=\frac{f_{0}}{\Delta f_{3\mathrm{dB}}}$$

where Δ*f*_3dB_ is the 3 dB bandwidth of |*S*_21_| in the simulation of the doubly loaded resonators and *f*_0_ is its center frequency [27]. The doubly loaded resonator is composed of a resonator, two irises, and two waveguide ports. Inductive irises with a width of 2.5 mm are employed to achieve weak coupling. Consider a doubly loaded TE_102_ mode resonator as an example, where all of the slots have a width of 1 mm and the equivalent length of the vertical slots on the side walls is 3 mm, as shown in Figure 4. Firstly, the metal model with no slots is simulated as a perfect electrical conductor (PEC), yielding *Q_e_* = *Q_L_* = 2.2531 × 10^4^, since there is no radiation or conductor loss and *Q_c_*^−1^ = *Q_r_*^−1^ = 0 in this case. Next, a PEC slotted model is simulated to obtain a *Q_L_*= 1.9159 × 10^4^ and *Q_r_* = (*Q_L_*^−1^ − *Q_e_*^−1^)^−1^ = 1.2800 × 10^5^, because *Q_c_*^−1^ = 0 for the same reason mentioned above. Finally, a slotted model with a copper boundary is simulated, yielding *Q_L_* = 4.2463 × 10^3^ and *Q_c_* = (*Q_L_*^−1^ − *Q_e_*^−1^ − *Q_r_*^−1^)^−1^ = 5.4555 × 10^3^. We can conclude that *Q_r_* is much higher than *Q_e_* and *Q_c_*, resulting in a much lower influence on *Q_L_*. *Q_u_* is calculated as 5.2325 × 10^3^ from Equation (4), which is close to the *Q_u_* (5.8721 × 10^3^) of the unslotted TE_102_ resonator. The above calculations demonstrate that the slots opened along the direction of the current have a negligible impact on the increase in insertion loss.

## 3. Design of the Filter

In this section, a sixth-order BPF centered at 20.4 GHz is designed, with a bandwidth of 1.6 GHz (FBW = 7.84%) and a return loss of 20 dB. Using the filter design methodology outlined in [27], coupling coefficients can be determined by the following equation:

(6) $$k_{\mathrm{ij}}=\frac{\mathrm{FBW}}{\sqrt{g_{i}g_{j}}}$$

where $g_{i}$ and $g_{j}$ are normalized element values of the lowpass prototype filter, while *i* and *j* refer to the *i*th and *j*th resonators. The inline topology is utilized to simplify the filter configuration and designing process, resulting in a matrix of [*k_ij_*]*_n_*_×*n*_, where *k_ij_* = 0 unless *i* = *j* ± 1, and *n* is the filter order. The matrix is listed as

(7) $${\left[k_{\mathrm{ij}}\right]}_{6\times 6}=\left[\begin{array}{cccccc} 0 & 0.0659 & 0 & 0 & 0 & 0\\ 0.0659 & 0 & 0.0479 & 0 & 0 & 0\\ 0 & 0.0479 & 0 & 0.0459 & 0 & 0\\ 0 & 0 & 0.0458 & 0 & 0.0479 & 0\\ 0 & 0 & 0 & 0.0479 & 0 & 0.0659\\ 0 & 0 & 0 & 0 & 0.0659 & 0 \end{array}\right]$$

And, the external quality factors are calculated as *Q_eS_* = *Q_eL_* = 12.71, based on the following equations:

(8) $$Q_{\mathrm{eS}}=\frac{g_{0}g_{1}}{\mathrm{FBW}}$$

(9) $$Q_{\mathrm{eL}}=\frac{g_{n}g_{n+1}}{\mathrm{FBW}}$$

**Figure 5:**
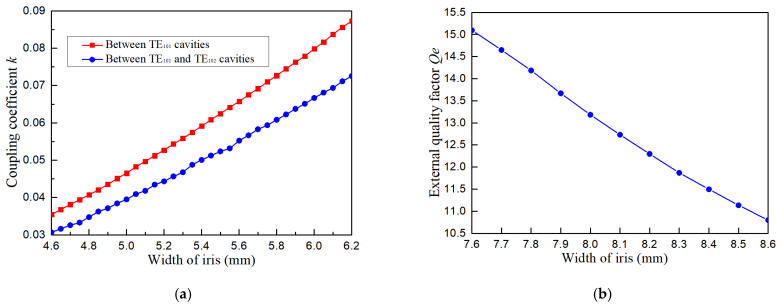
Extraction of the width of an iris: (**a**) coupling coefficient *k* against the width of the iris; (**b**) external quality factor *Q_e_* against the width of the iris.

Inductive irises are utilized between resonators and at ports. The curves of coupling coefficient *k* and external quality factor *Q_e_* against the width of the iris are pictured as Figure 5, from which the initial values of the irises are extracted. It is noticed that the TE_102_ cavity cascaded to the filter port to obtain *Q_e_* in Figure 5b.

**Figure 6:**
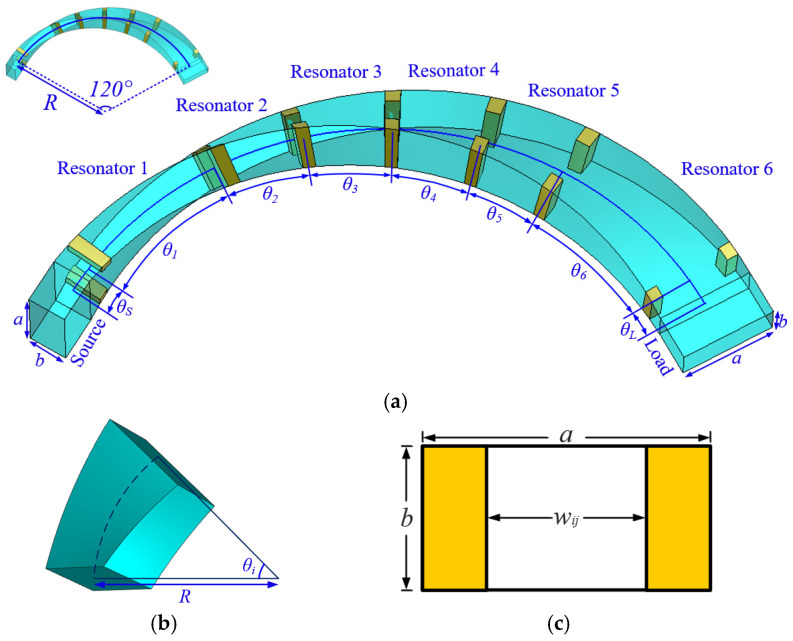
Internal configuration of the proposed BPF: (**a**) the overall configuration; (**b**) vacuum box mode of a distorted rectangular waveguide resonator; (**c**) structure of an inductive iris.

The internal configuration of the proposed BPF is displayed in Figure 6, consisting of six distorted rectangular waveguide resonators, seven pairs of inductive irises, and two rectangular waveguide ports. Resonator 1 and 6 are TE_102_ resonators, which have a significantly longer length compared to the other TE_101_ resonators. Standard WR-42 waveguides are utilized as input and output ports, with a cross-section of 10.668 mm × 4.318 mm. A 90° polarization rotation is achieved between the two waveguide ports by applying a 120° turn to the waveguide path in the filter body, while the bending radius is *R* = 38 mm. All of the distorted inductive irises have an equivalent thickness of 1.5 mm. Optimization is required to obtain the exact values for all parameters, as listed in Table 1.

**Figure 7:**
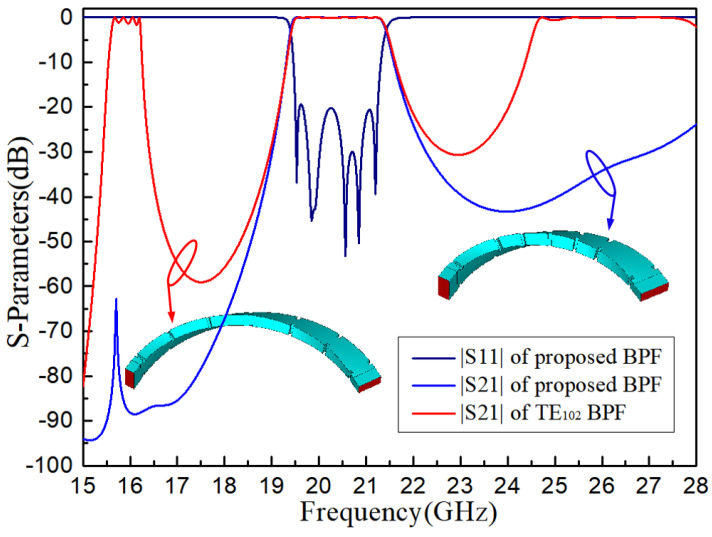
S-parameters of the proposed BPF compared with the TE_102_ BPF.

To elaborate on the advantages of the stopband performance of the proposed BPF, a filter using only TE_102_ cavities with the same indexes is simulated. The parameters of |S_21_| are pictured in Figure 7 for comparison, which notes better stopband suppression of the proposed BPF. This is because the harmonic responses of the TE_102_ BPF are determined by its longer TE_102_ mode resonators; however, those of the proposed PBF are mainly determined by the TE_101_ mode resonators, which have wider frequency gaps between the resonating frequencies of the TE_10n_ modes.

## 4. Fabrication, Measurement, and Discussion

Three-dimensional printing technology was utilized to fabricate the filter prototype. It follows an additive manufacturing process and prints the structure as a monolithic piece. According to the properties of raw materials, 3D printing can be categorized into metal printing and non-metal printing. SLA is one of the most widely used techniques of non-metal printing for RF waveguide devices, relying on the photopolymerization of liquid photosensitive resin with high precision. The SLA process involves the following steps: (a) importing the CAD model into the SLA printer and slicing it into layers; (b) positioning the platform in a tank filled with liquid photosensitive resin, and using ultraviolet laser to cure the current layer; and (c) sequentially curing and printing each layer as the platform moves, ultimately forming the complete model. It is important to note that photopolymerization is an irreversible process, meaning that the solid resin model cannot be reverted to its liquid state by heating.

Then, a metallization process of the model surface is essential to equip the resin model with excellent electrical conductivity and electromagnetic performance comparable to that of metal devices. Metallization is typically accomplished through electroless nickel plating followed by electroplating with copper (or silver, gold, etc.). The model surface is firstly activated using plasma irradiation and chemical solutions to enhance its ability to adsorb metal particles. Subsequently, a 1 μm nickel layer is electrolessly plated onto the surface, followed by a 10 μm copper layer using electroplating. It should be emphasized that the copper layer thickness is significantly greater than the skin depth at the filter’s center frequency, yet not so thick as to cause blistering in the copper electroplating.

**Figure 8:**
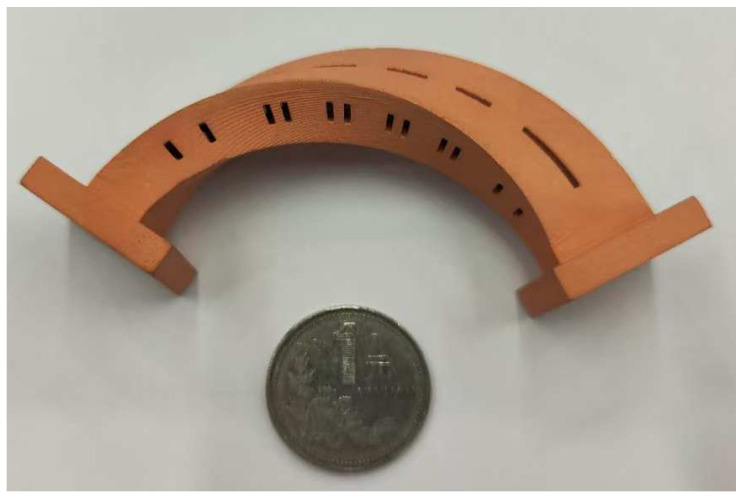
A photograph of the proposed BPF.

These steps result in the fabrication of the filter prototype. A photograph of the filter is shown in Figure 8. A few rectangular slots are positioned on the cavity walls, for the ease of plating and with little radiation loss, as analyzed earlier. The overall size of the filter is 92.23 mm × 31.33 mm × 22.41 mm (9.10*λ_g_* × 3.09*λ_g_* × 2.21*λ_g_*), where *λ_g_* is the waveguide wavelength at center frequency in the passband.

**Figure 9:**
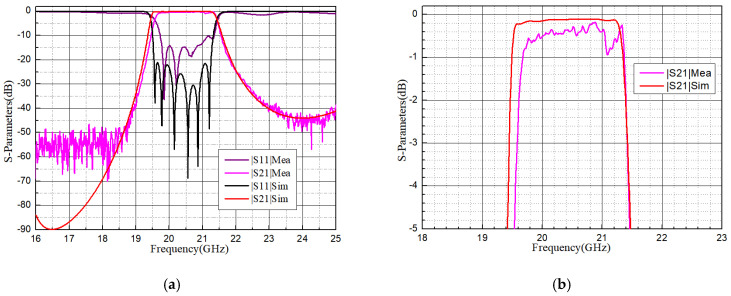
Measurements compared with simulations: (**a**) overall S-parameters; (**b**) |S_21_| in detail.

Measured results were obtained by a network analyzer, and the S-parameters are pictured in Figure 9, compared with simulations. A shift in the center frequency (Δ*f*_0_) of about 100 MHz (0.5%) toward higher frequencies can be observed. The return loss (RL) in the passband is better than 10.37 dB. And, the insertion loss (IL) in the passband is about 0.31 dB on average and lower than 0.94 dB, while the simulated IL is about 0.46 dB. In general, the measurements show good consistency with the simulations.

Discrepancies between the simulations and measurements primarily manifested in three aspects: (a) deterioration of return loss; (b) deterioration of insertion loss; and (c) frequency shift. Firstly, the deterioration of return loss is mainly attributed to the errors introduced during manufacturing. Minor variations in the width of irises have a significant impact on return loss and lead to a reduction in the number of transmission poles. It should be noted that the reduction in transmission poles does not indicate that some resonators are malfunctioning, as the number of resonators, that is, the order, affects the filter’s performance not only in terms of return loss but also in terms of the attenuation rate of |S_21_| outside the passband. Secondly, the deterioration of insertion loss originates from the roughness of the copper layers, which induces additional reflection and absorption of the electromagnetic fields and is difficult to measure directly. An intuitive method to evaluate the roughness is to observe whether the filter’s surface is flat and whether there are obvious bulges. Lastly, frequency shift is mainly caused by a little shrinkage in the whole filter body during the cooling process after 3D printing and before metal plating, which causes the filter’s passband to move to higher frequencies.

Finally, some other 3D-printed waveguide BPFs integrated with bending and/or twisting functions are listed in Table 2, in comparison with the proposed BPF in this paper. Shape factor is calculated as the ratio of the 3 dB bandwidth to the 40 dB bandwidth. A larger shape factor means greater selectivity of the BPF. It is noticed that an obviously lower insertion loss and greater selectivity are achieved in this work, with a novel 120° bend in the waveguide routine.

## 5. Conclusions

In this paper, a sixth-order waveguide BPF employing mixed TE_101_ and TE_102_ modes and integrated with 90° twisting and 120° bending is proposed. The filter demonstrates lower insertion loss compared to conventional TE_101_ mode BPFs and superior stopband performance relative to TE_102_ mode BPFs. A filter prototype is designed at a center frequency of 20.4 GHz, with a bandwidth of 1.6 GHz (FBW = 7.84%) and a return loss of 20 dB. SLA technology is utilized in the fabrication process, and a plating process provides the filter with excellent electrical properties. The measured insertion loss is about 0.46 dB on average and lower than 0.94 dB, while return loss is better than 10.37 dB. A shift in the center frequency of about 0.5% is observed as well. The proposed filter, integrated with bending and twisting functions, can effectively reduce the overall size of the device and minimize losses caused by misalignment in traditional multi-component cascaded designs.

## Figures and Tables

**Table 1:** Exacted values of all parameters after optimization.

Parameters	Values (mm)	Parameters	Values (°)
*a*	10.668	*θ_S_*	4.368
*b*	4.318	*θ*_1_	26.823
*w_S_*_1_	7.877	*θ*_2_	13.986
*w*_12_	5.841	*θ*_3_	14.823
*w*_23_	4.962	*θ*_4_	14.823
*w*_34_	4.859	*θ*_5_	13.986
*w*_45_	4.962	*θ*_6_	26.823
*w*_56_	5.841	*θ_L_*	4.368
*w*_6*L*_	7.877		

**Table 2:** Other 3D-printed waveguide BPFs integrated with bending and/or twisting functions compared with the proposed BPF in this paper.

Ref.	Tech.	Clad Layer	*f*_0_ (GHz)	FBW (%)	Resonator Shape	Mode	Rotation of Polarization (°)	Waveguide Bending (°)	IL (dB)	Shape Factor
[16]	SLA	1 μm Silver and 10 μm Copper	32	3.125	Rectangular	TE_101_	90	None	0.84 on average	~0.31
[17]	SLA	1 μm Silver and 10 μm Copper	34	2.9	Rectangular	TE_102_	90	None	0.81 on average	~0.32
[18]	SLA	10 μm Copper	15	4	Rectangular	TE_101_	90	90	0.85 on average	~0.33
This work	SLA	10 μm Copper	20.4	7.84	Rectangular	TE_101_ and TE_102_	90	120	0.46 on average	~0.35

## Data Availability

The data are contained within this article.
